# Developmental regression of novel space preference in an autism spectrum disorder model is unlinked to GABAergic and social circuitry

**DOI:** 10.3389/fncel.2024.1513347

**Published:** 2025-01-15

**Authors:** Hirofumi Asano, Masaya Arai, Aito Narita, Takayuki Kuroiwa, Mamoru Fukuchi, Yuhei Yoshimoto, Soichi Oya, Goichi Miyoshi

**Affiliations:** ^1^Department of Developmental Genetics and Behavioral Neuroscience, Gunma University Graduate School of Medicine, Maebashi, Gunma, Japan; ^2^Department of Neurosurgery, Gunma University Graduate School of Medicine, Maebashi, Gunma, Japan; ^3^Laboratory of Molecular Neuroscience, Faculty of Pharmacy, Takasaki University of Health and Welfare, Takasaki, Gunma, Japan

**Keywords:** GABAergic development, ASD model, novel space preference, regression, social behavior

## Abstract

Autism spectrum disorder (ASD) is characterized by social deficits and restricted behaviors, with developmental defects in GABAergic circuits proposed as a key underlying etiology. Here, we introduce the V-Y assay, a novel space preference test in which one arm of the Y-maze is initially hidden and later revealed as a novel space. Using an ASD mouse model with *FOXG1* haploinsufficiency, which exhibits ASD-like social impairments that can be either exacerbated or ameliorated by GABAergic circuit manipulations, we observed impaired novel space preference and exploratory behavior in the V-Y assay. Interestingly, unlike social phenotypes, novel space preference was initially established by 3 weeks of age but regressed by 6 weeks. Furthermore, alterations in GABAergic signaling via *Gad2* mutation did not affect novel space preference, in contrast to their impact on social behaviors. These findings reveal that the regression of novel space preference in ASD follows a distinct developmental trajectory from GABA-driven social impairments, providing new insights into the mechanisms underlying ASD.

## Introduction

Autism spectrum disorder (ASD) is characterized by social communication deficits and restricted, repetitive behaviors (DSM-5), with a prevalence of 1–2% among children (CDC, United States). Abnormal inhibition mediated by cortical GABAergic interneurons has been implicated in ASD etiology. Postmortem studies of ASD patients have shown a loss of inhibitory neurons (Hashemi et al., 2017), and epilepsy is a common comorbidity in ASD. Moreover, ASD mouse models with conditional mutations in syndromic ASD genes within GABAergic populations often reproduce the behavioral phenotypes observed in straight-null animals (Chao et al., 2010; Judson et al., 2016). These findings support the current hypothesis that disruptions in GABAergic signaling are central to ASD pathology (Nelson and Valakh, 2015; Pizzarelli and Cherubini, 2011; Rubenstein and Merzenich, 2003).

In addition to core symptoms such as social and repetitive behavioral impairments, ASD patients often display deficits in spatial recognition. They tend to learn spatial regularities and locations more slowly, relying on allocentric representations. These individuals also show reduced novel space preference, are less likely to explore environments thoroughly, and more likely to revisit previously explored locations (Smith, 2015). While visuospatial abilities are considered a strength in some ASD patients (Mottron et al., 2006; Stevenson and Gernsbacher, 2013), a growing body of evidence points to challenges in spatial processing (Bochynska et al., 2020).

Rett syndrome, caused by mutations in the *Mecp2* gene on the X chromosome, is classified as a syndromic form of ASD (Sztainberg and Zoghbi, 2016). In Rett syndrome, while the extent of X-inactivation in the nervous system of girls contributes to the severity of the disease, developmental regression is observed after a period of seemingly normal motor, cognitive, and social development in early infancy, followed by a severe loss of abilities around 1–2 years of age. Regression phenotypes are also observed in female mouse models of Rett syndrome (Mykins et al., 2024). A significant subgroup of ASD children also experience developmental regression, particularly in language and social communication (Williams et al., 2015), with approximately one-third losing previously acquired skills during their second year of life (Boterberg et al., 2019; Tammimies, 2019). Despite these observations, the relationship between GABAergic neuron development and the loss of previously acquired skills in ASD remains poorly understood.

To better understand ASD etiology and explore potential treatments, numerous transgenic mouse models have been developed to study social behavior impairments (Chao et al., 2010; Judson et al., 2016; Miyoshi et al., 2021; Nakatani et al., 2009; Peca et al., 2011; Schmeisser et al., 2012; Silverman et al., 2010). Spatial recognition deficits have also been studied in ASD models (Arbab et al., 2018; Kleijer et al., 2018), along with regression phenotypes (Kshetri et al., 2024). Our previous work focused on *FOXG1*, a transcription factor strongly associated with ASD (Mariani et al., 2015) and involved in GABAergic neurogenesis (Miyoshi et al., 2024). *FOXG1* dysregulation during development has been proposed as an endophenotype of idiopathic ASD, supported by patient iPS cell-derived brain organoids (Mariani et al., 2015). Both haploinsufficiency and gene duplication of *FOXG1* lead to the development of FOXG1 syndrome, classified to ASD (Brimble et al., 2023). The significance of precise *FOXG1* gene dosage is highlighted by its dynamic expression changes in migrating neuronal precursors, which are crucial for cortical circuit formation (Miyoshi and Fishell, 2012; Miyoshi et al., 2024). We recapitulated human *FOXG1* phenotypes by decreasing or increasing *FoxG1* levels in mouse neurons, thereby creating *FOXG1* ASD mouse models. Our findings highlight a critical developmental period during early juvenile stages in the emergence of ASD-related social impairments. Furthermore, we show that these social behaviors can be either exacerbated or ameliorated depending on the timing of GABAergic circuit manipulation (Miyoshi et al., 2021).

In this study, we modified the Y-maze (Hellyer and Straughan, 1961) to develop the V-Y maze, specifically designed to detect novel space preference in model mice. We found that the *FOXG1* haploinsufficiency ASD mouse model transiently forms a novel space preference during early juvenile stages, but this preference regresses by 6 weeks of age. Unlike social behavior impairments, which are highly dependent on GABAergic circuit development, the regression of novel space preference occurs independently of this pathway. Our findings highlight the distinct developmental trajectories of spatial recognition and social behaviors in ASD, shedding light on the differential role of the GABAergic system in these phenotypes.

## Materials and methods

### Animal experiments

All animal handling and experiments were performed in accordance with protocols approved by the Institutional Animal Care and Use Committees of the Gunma University Graduate School of Medicine. Animal cages are maintained at 22°C ± 1°C, 50 ± 15% humidity, with a 12-h light/dark cycle. ALPHA-dri bedding (Shepherd, Technical grade # L-2307-1178 AD06123) and a pellet diet (Rodent Diet CE-2, gamma irradiated, CLEA) were used to maintain the mouse colony. After mating, the morning plug observed was considered embryonic day 0. Pups were typically delivered on embryonic day 19, corresponding to postnatal day 0 (P0). We conducted behavioral experiments in male mice; therefore, female pups were removed at P0. The dam was kept in either a small (W140 × D320 × H140mm, KN-60105-TPX, Natsume Seisakusho) or medium-sized cage (W215.8 × D316.8 × H150mm, KN-600U-TPX, Natsume Seisakusho). When pups reached postnatal day 14 (P14), the entire litter was placed in a medium-sized cage with a few pellet diets on the floor. At postnatal day 21, the whole litter was weaned and placed into a larger cage (W270 × D440 × H187mm, KN-601-TPX, Natsume Seisakusho). Genotyping of the animals by tail PCR was typically performed by postnatal week 2. After completing battery of behavioral analyses, tail PCR was repeated to assure the genotypes.

The ASD model and the control wildtype littermate animals were generated by crossing a male mouse heterozygous for the *FoxG1 LacZ* knock-in null allele (Xuan et al., 1995) with a wildtype female. A small proportion of ASD model mice exhibited spinning behavior in the home cage and were therefore excluded from the behavioral study. Pups were genotyped using PCR with three primers: FoxG1 10960F (AAGGGCAACTACTGGATGCTCGAC), Neo 1531F (TTGAATGGAAGGATTGGAGCTAC) and FoxG1 11611R (ACAGTCCTGTCGTAAAACTTGGC), which produced wildtype (652 bp) and mutant (~400 bp) bands (Miyoshi et al., 2021). We reduced GABAergic tone during development by utilizing a mutant allele of *Gad2*, the enzyme responsible for GABA synthesis. To perform littermate studies for *FoxG1*; *Gad2* compound mutants, double-heterozygous male *FoxG1-LacZ*; *Gad2-null* animals were crossed with female *Gad2-null* heterozygotes (Yanagawa et al., 1999). Pups carrying the *Gad2-null* heterozygous allele were removed after genotyping (Miyoshi et al., 2021).

### Behavioral assays

V-Y assays for 2-, 3-, and 6-week-old male mice were conducted on independent sets of animals at P15–P17, P22–P24, and P43–P45, respectively. The three-chamber assay was performed on P21 and P42 for the 3-and 6-week-old mice, respectively, prior to the V-Y assay. Behavioral assays were conducted in a soundproof room (S-1520 DX, STAR LITE), with mouse behavior in each specific arena recorded by a Progressive Scan CMOS camera (USB 3.1 Blackfly S, Monochrome Camera, BFS-U3-51S5-C, FLIR) at 15 frames per second and saved as M-JPEG files with 75% compression using Spinnaker camera software 2.7.0.128 (FLIR). Prior to recording, the test animal’s information—such as the date of filming, animal number, and presence or absence of a social animal—was written on a small whiteboard (24 × 30 cm) and placed in the testing area. After video recording began, the whiteboard was removed, and the test animal was placed in the arena, or the starting dome was removed to release the animal. Once testing commenced, the experimenter quickly left the soundproof chamber and quietly closed the door to provide an undisturbed environment during the assay. After completing the video recording, the test animal was returned to a new cage containing previously tested littermate animals. The experimenter was always blind to the genotype of the test animals. Video files were later analyzed using ANY-maze video tracking software 7.4 (Stoelting, United States), with video analysis typically starting after the door was closed. All behavioral analysis data are presented as mean ± SEM.

### V-Y maze novel space preference task

**Figure 1:**
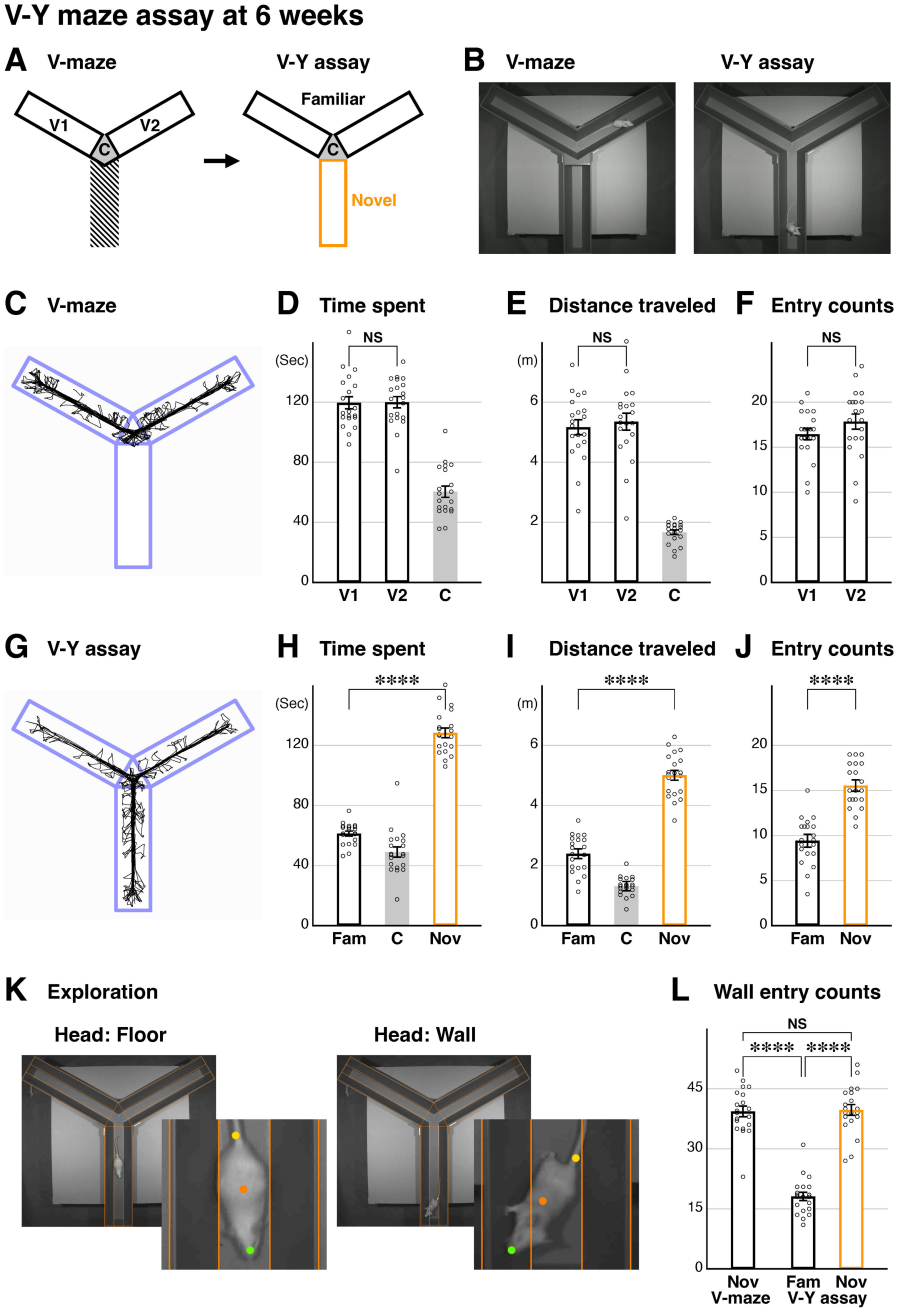
Development of the V-Y maze assay for analyzing novel space preference and exploratory behavior in mice. **(A)** A schematic of the V-Y maze assay. During the initial V-maze phase, arms V1, V2, and the center (C) are analyzed separately. In the subsequent V-Y maze phase, the previous V1 and V2 arms become familiar arms, while a newly introduced arm becomes the novel arm. **(B)** Representative video images from the V-maze and V-Y assays at 6 weeks (P43-45). A custom movable wall block was designed to create continuous V-shaped arms in the center region. **(C–F)** A representative behavioral trace of the test animal’s body **(C)**. Analysis of the V-maze phase (*n* = 20). Time spent (**D**, *p* = 0.950), distance traveled (**E**, *p* = 0.623), and entries (**F**, *p* = 0.186) in the V1 and V2 arms were comparable. **(G–J)** A representative behavioral trace of the test animal’s body during the V-Y assay phase **(G)**. Analysis during the V-Y phase (*n* = 20). Time spent [**H**, *p* = 2.78×10(−17)****], distance traveled [**I**, *p* = 1.52×10(−14)****], and entries [**J**, *p* = 3.46×10(−9)****] in the novel arm were significantly increased compared to the familiar arms (average of V1 and V2). **(K,L)** Exploratory behavior of the test animals was analyzed based on head positions (**K**, green dots), and the entries into the walls are presented **(L)**. Note that the central regions of the wall at the end of each arm were not included in the analysis **(K)**. Higher magnification views of the body and head positions are also shown **(K)**. While entries into the wall domain were comparable between the novel arms of the V-maze and V-Y assays (*p* = 0.768), both were significantly increased compared to the familiar arms [*p* = 2.07×10(−12)**** for V-maze and *p* = 9.78×10(−15)**** for V-Y assay], suggesting that the mice are actively exploring the novel arm. Data are mean ± SEM, *p-*values are from two-tailed *t*-tests, except for the V and V-Y comparison in **(L)**, which is from a paired two-tailed *t*-test.

The Y-maze (YM-03M, Muromachi) consists of a 40 mm triangular center region with each arm measuring 40 mm wide floor and 415 mm in length. The top of the wall is 100 mm wide, and the vertical height is 100 mm. Both the Y-maze and the movable wall block used for the V-maze assay are made from gray vinyl chloride. A video recording was initiated for 11 min at a resolution of 2,200 × 1,948, with a frame rate of 15 frames per second. After the whiteboard was removed and the test animal was placed at the end of the V2 arm, the experimenter quickly and quietly left the soundproof chamber and closed the door. After 5 min and 30 s of recording, the experimenter quickly entered the soundproof chamber, removed the movable wall unit to allow access to all three arms of the Y-maze (V-Y assay), and then exited the chamber quietly, closing the door. Data were analyzed over the 5-min session, measuring time spent, distance traveled, and entry counts for the arms and center based on body location. We used identical analysis methods for the V-maze and V-Y assays in the Any-maze software. For the center region of the V-maze, small portions of the third arm region adjacent to the center area were also included in the analysis (see the scheme for the center region in Figure 1A). For entry counts into the arms, transitions such as V1-Center-V1 and V2-Center-V1 were both counted as a single entry event into the V1 arm. Additionally, entry counts into the wall or floor of each arm were analyzed based on head position. Note that, at the end of each arm, the wall regions with a width equal to that of the floor were not considered walls in our analysis (Figure 1K). For the 2-week-old V-Y assay, 2 wildtype and 6 ASD model animals that did not leave the V2 start arm during the V-maze session were excluded from the analysis. Similarly, 2 *Gad2* null ASD model animals were excluded because they remained in the start arm during the V-maze assay.

### Three-chamber social interaction assay

The apparatus consists of a gray acrylic-modified polyvinyl-chloride floor and three chambers (each 20 cm × 40 cm) connected by 5 cm wide × 7 cm high windows in transparent acrylic walls, 22 cm in height (SC-03M, Muromachi). The wire cages are 18.5 cm high with a 9 cm diameter circular base and top, connected by 16 wires, each 3 mm in diameter, placed in a circle with a 7 mm gap between them. Of the four wire cages, two were used to hold a stranger mouse, while the other two remained empty for the Habituation phase (first 10 min) and the non-social side of the Sociability session (second 10 min). The chambers and wire cages were cleaned and dried with paper towels between each test animal trial.

The test mouse was initially placed in a start dome (20 cm diameter circular transparent acrylic tube) in the middle chamber. A video recording was started at a resolution of 1,900 × 1,300, with a frame rate of 15 frames per second. Once the whiteboard and dome were removed, the experimenter quickly left the soundproof room, quietly closing the door. During the first session (Habituation), the animal was allowed to freely explore the three chambers, with two empty wire cages placed in the center of the lateral chambers. After 10 min and 30 s of video recording, the test animal was returned to the middle chamber and trapped in the start dome. In the second session (sociability), one of the empty wire cages was replaced with a cage containing an age-matched stranger mouse. Another 10 min and 30 s of video recording was obtained. The test animal was then again confined to the start dome. In the final session (Social Novelty), the remaining empty cage was replaced with a cage containing another stranger mouse. While 6-week-old animals normally do not climb on top of the wire cages, 3-week-old animals did. Therefore, we excluded animals that climbed on top of the wire cages (WT: 17 and ASD model: 3) from our analyses.

## Results

### The V-Y arm maze assay reveals a novel space preference and exploratory behavior

The Y-maze assay, which consistently presents two arm choices to test animals, is often used for analyzing repetitive entry behavior into the same arms. To evaluate whether a test mouse prefers to enter a newly revealed arm, we initially hid one arm of the Y-maze and then revealed it during the late phase of the assay (Figure 1A). To hide a single arm, we used specific movable walls (Figure 1B), which could later be removed to display three arm choices. We analyzed the behavior of 6-week-old (6w) mice over a 5-min period during the V-maze phase (Figure 1C), which consisted of two arms, to assess differences in time spent (Figure 1D), distance traveled (Figure 1E), and entry counts (Figure 1F) between the two arms (V1 and V2) (see schematic in Figure 1A). The analysis indicated no significant differences between V1 and V2 arms in these metrics. Upon removing the movable walls for the Y-maze phase (Figure 1G), we compared the familiar V arms (V1 and V2, averaged) with the newly revealed novel arm over a 5-min period (Figures 1A,B). We found that the test animals showed an increase in time spent, distance traveled, and number of entries in the novel arm compared to the familiar arms (Figures 1H–J). This strongly suggests that our V-Y assay effectively demonstrates a preference for novel space in the animals.

Next, using the head position of the animals (Figure 1K, green dots), we assessed whether the animals preferred to stay on the floor or stand against the wall during the assay. Crossing of the head position from the floor into the wall domain (Figure 1K) were counted as wall entries (Figure 1L). We found that wall entry counts were comparable during both the initial V-maze phase and the novel arm of the V-Y assay (Figure 1L). However, wall entries were significantly decreased in the familiar arms of the V-Y assay compared to both the V-maze arms and the novel arm of the V-Y assay (Figure 1L). This suggests that when animals explore a novel environment, they tend to spend more time seeking the walls. In conclusion, our V-Y maze assay highlights a preference for novel space in animals, as indicated by increased time spent in the novel arm and enhanced wall-seeking exploratory behavior.

### Both novel space preference and exploratory behavior are attenuated in the ASD mouse model

**Figure 2:**
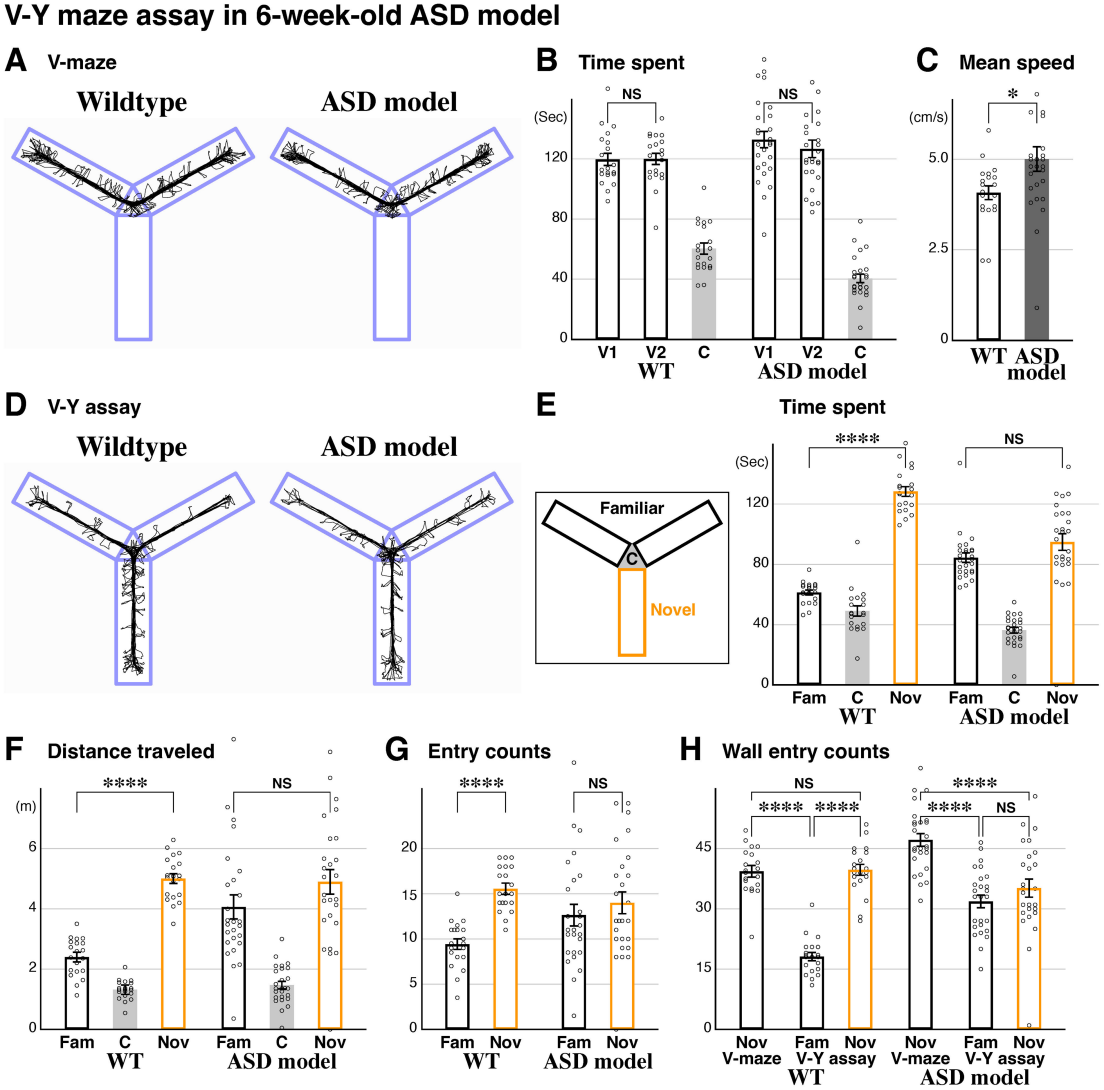
Autism spectrum disorder (ASD) model displays impairments in novel space preference and explorative behavior. The V-maze assay followed by the V-Y maze assay was carried out in littermate wildtype (*n* = 20) and ASD model (*FoxG1* heterozygous, *n* = 26) mice at postnatal 6 weeks (P43-45). **(A)** Representative traces of the two mouse models. **(B,C)** During the V-maze assay, the time spent in the V1 and V2 arms was comparable within each model (**B**, *p* = 0.445, ASD model). Mean speed was increased in the ASD model compared to wildtype animals (**C**, *p* = 0.0216*). **(D)** Representative traces during the V-Y assay. **(E–G)** While time spent, distance traveled, and entries in the novel arm were increased compared to the familiar arms in wildtype animals (same data as in Figures 1H–J), this was not the case in the ASD model (*p* = 0.110, 0.142, and 0.426, respectively). **(H)** Explorative behavior was analyzed based on head positions. Unlike the wildtype littermates (Figure 1L), the ASD model exhibited comparable wall entry counts between the familiar and novel arms during the V-Y assay (*p* = 0.232) and showed a significant decrease in the novel arm during the V-Y assay compared to the V-maze [*p* = 1.32×10(−5)****]. Additionally, wall entry counts in the V arms were significantly reduced during the V-Y assay in the ASD model animals [*p* = 6.87×10(−11)****]. These data suggest that the ASD model exhibits overall alterations in explorative behavior. The raw data points for 222.7 (**B**, V2, ASD), 7.9 and 10.5 (**C**, ASD) are not shown. Data are mean ± SEM, *p-*values are from two-tailed *t*-tests, except for the V and V-Y comparisons in **(H)**, which are from paired two-tailed *t*-tests.

After establishing our V-Y assay, we investigated novel space preference in a mouse model related to ASD. A heterozygous mouse for the transcription factor *FoxG1* (Xuan et al., 1995) shows impaired social behavior, reduced gamma frequency EEG power in the prefrontal cortex, and has been characterized as an ASD mouse model (Miyoshi et al., 2021). We thus utilized 6-week-old *FoxG1-LacZ* heterozygous ASD model animals and compared them with littermate wildtype (WT) controls. During the initial V-maze phase (Figure 2A), the time spent in the two V arms was comparable within both the wildtype and ASD model groups (Figure 2B). However, the mean speed, measured based on body position, was increased in the ASD model compared to the control wildtypes (Figure 2C). In the V-Y assay phase (Figure 2D), which investigates novel space preference, we found that, unlike the control littermates, the time spent, distance traveled, and number of entries for the novel arm were comparable to the familiar arms in the ASD model (Figures 2E–G). We observed a similar trend in mean speed during the V and V-Y assays (Figure 2C, and data not shown). This strongly suggests that the ASD model does not show a novel space preference, in addition to impairments in social behavior (Miyoshi et al., 2021). Consistent with this finding, when exploratory behavior of the ASD model was analyzed, we found that head entries into the wall domain were comparable between the familiar and novel arms during the V-Y assay phase (Figure 2H). We conclude that the ASD model demonstrates a lack of interest in novel space, even though it has been shown to display spatial preference (Narita et al., 2024).

### The ASD model acquires novel space preference during the juvenile stage and subsequently regresses during development

**Figure 3:**
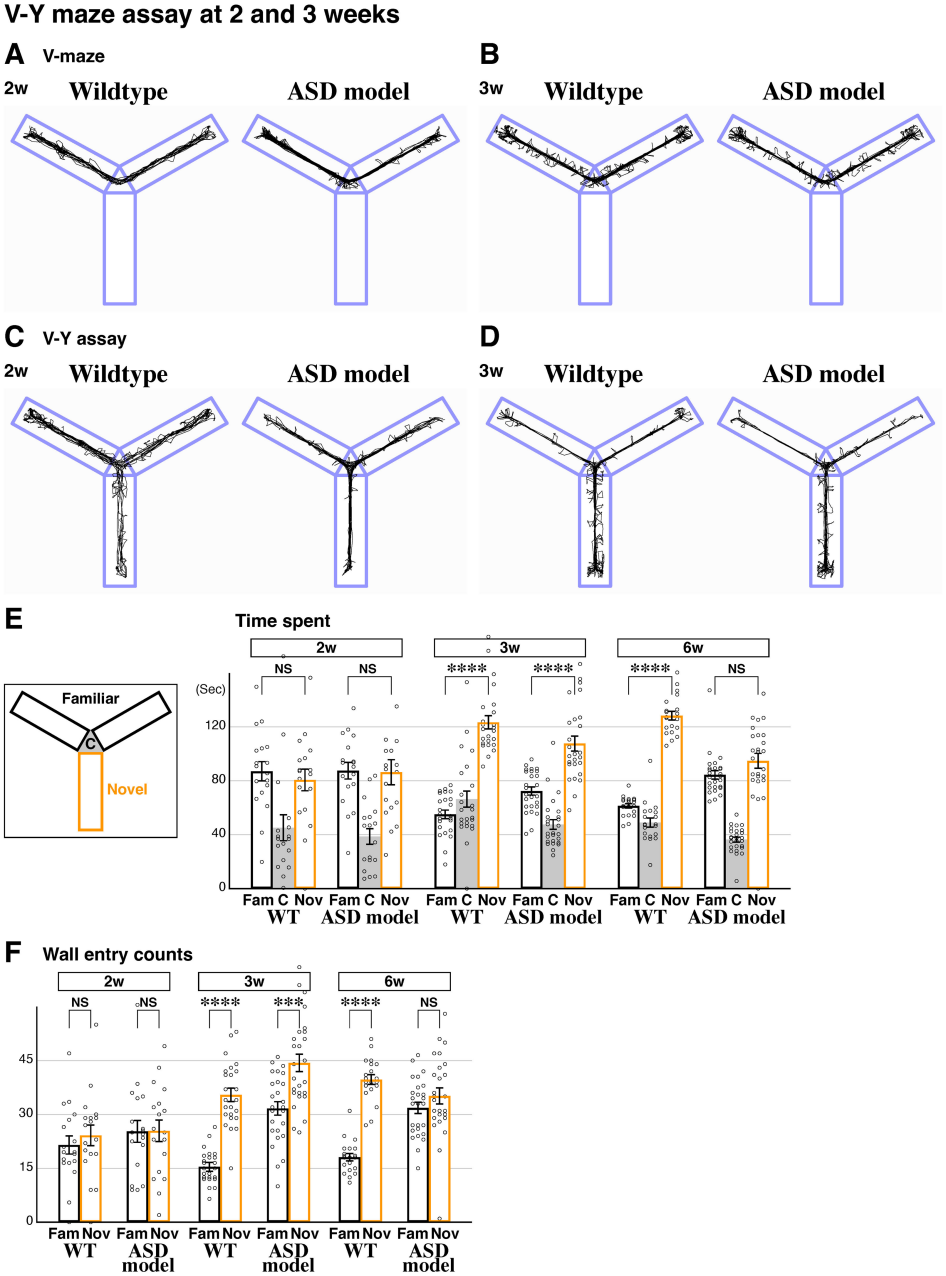
Developmental regression of novel space preference in the ASD model. **(A–D)** Representative behavioral traces of littermate wildtype and ASD model (*FoxG1* heterozygous) mice during the V-maze assay **(A,B)** and V-Y assay **(C,D)** at 2 (P15-17, **A,C**) and 3 weeks (P22-24, **B,D**). **(E)** Time spent in each arm during the V-Y assay. At postnatal 2 weeks, time spent in the familiar and novel arms was comparable in both wildtype (*n* = 18, *p* = 0.553) and ASD models (*n* = 18, *p* = 0.919), suggesting that novel space preference had not yet developed in either model at this age. At postnatal 3 weeks, unlike at 2 weeks, both models spent significantly more time in the novel arm compared to the familiar arms [*n* = 24, *p* = 2.12×10(−14)**** for wildtype and *n* = 26, *p* = 1.97×10(−6)**** for ASD]. At 6 weeks (P43-45), the ASD model no longer showed a preference for the novel arm (data from Figure 2E), indicating a regression of novel space preference by 6 weeks in the ASD model. **(F)** In 6-week-old wildtype mice, the number of head position entries into the wall was higher in the novel arm compared to the familiar arms (data from Figure 1L). This was similarly observed at 3 weeks [*p* = 3.36×10(−11)****] but not at 2 weeks (*p* = 0.489), indicating that exploratory behavior typically becomes evident by postnatal 3 weeks. However, in the ASD model, wall entries in the novel arm compared to the familiar arms increased only at 3 weeks [*p* = 1.50×10(−4)***] but not at 2 (*p* = 0.969) or 6 weeks (data from Figure 2H). This trend was similar to the time spent in each arm **(E)**, suggesting that the ASD model starts to exhibit defects in exploratory behavior after 3 weeks. The raw data point 198.8 (**E**, 2w, Nov, ASD) is not shown. Data are mean ± SEM, *p-*values are from two-tailed *t*-tests.

To understand the developmental process for the acquisition of novel space preference, we performed our V-Y maze assay at 2 weeks (P15-17, Figures 3A,C) and 3 weeks (P22-24, Figures 3B,D), in addition to the analysis conducted at 6 weeks of age (Figure 2). At 2 weeks, the time spent in the familiar and novel arms was comparable in wildtype animals (Figure 3E). However, at 3 weeks, the time spent in the novel arm was significantly increased compared to the familiar arm (Figure 3E). These data suggest that mice generally acquire novel space preference during juvenile developmental stages between 2 and 3 weeks and maintain it through 6 weeks (Figures 1H, 3E) and into adulthood. In the ASD model, we found that the time spent in the familiar and novel arms at 2 weeks was comparable, similar to littermate wildtype controls (Figure 3E). However, at 3 weeks, the time spent in the novel arm was significantly increased compared to the familiar arm, strongly suggesting that the ASD model acquires novel space preference between postnatal 2–3 weeks (Figure 3E). Later, at 6 weeks, the ASD model does not show a preference for novel space (Figures 2E, 3E). These data indicate that the ASD model develops a preference for novel space by postnatal 3 weeks, similar to wildtype animals, but subsequently loses this preference by 6 weeks of age.

We next analyzed wall entry counts during the V-Y assay to investigate the exploratory behavior of the animals. At 2 weeks, both wildtype and ASD model displayed comparable entries between the familiar and novel arms (Figure 3F). However, by 3 weeks, both models showed an increase in wall entry counts in the novel arm compared to the familiar arms (Figure 3F). Since wall entries in the ASD model become comparable between the familiar and novel arms by 6 weeks (Figures 2H, 3F), the exploratory behavior of the ASD model appears to be transiently established by postnatal 3 weeks and subsequently regresses during development. Altogether, we conclude that novel space preference is acquired between postnatal weeks 2 and 3. In the ASD model, this preference is initially transiently established but subsequently regresses, disappearing by 6 weeks of age.

### The regression of spatial preference in the ASD model occurs independently of GABAergic neuron development

**Figure 4:**
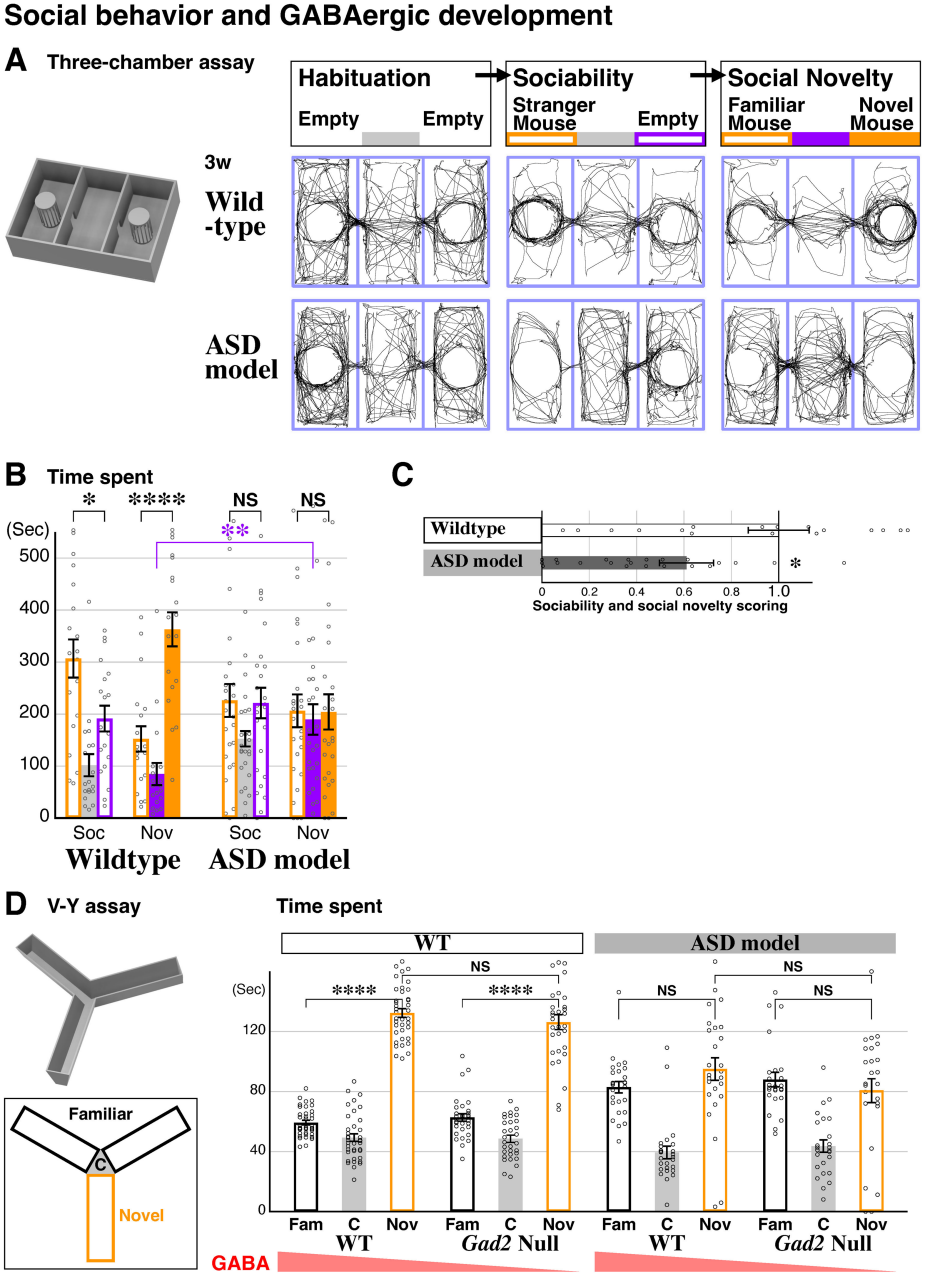
Novel space preference is formed independent of social and GABAergic pathways. **(A)** A 3-D model of the three-chamber social assay setup, and representative traces of 3-week-old (P21) animal body location are shown for both wildtype and *FoxG1* heterozygous (*LacZ* knock-in) littermates during each 10-min session of Habituation, Sociability, and Social Novelty. **(B,C)** Social behavior of the animals was analyzed by comparing the time spent in each chamber of the three-chamber assay. Wildtype animals preferred to spend time in the social side of the chambers (**B**, orange bar graphs), whereas ASD model animals did not exhibit a social preference (**B**, NS for left vs. right chamber). WT: *n* = 19, *p* = 0.0136*(Soc), *p* = 1.12×10(−5)****(Nov), Het: *n* = 25, *p* = 0.908(Soc), *p* = 0.962(Nov). Additionally, the ASD model preferred the middle chamber (filled purple bar graph, *p* = 0.00620**) during the social novelty session and avoided the two lateral chambers containing other mice. Social behavior scores **(C)** are calculated based on the time spent in the social side (orange bars in **B**) of the chambers (*p* = 0.0295*). **(D)** The V-Y maze assay was performed on 6-week-old (P43–45) littermate wildtype (*n* = 38), *Gad2* null (*n* = 31), *FoxG1* heterozygous (*n* = 26), and *Gad2* null; *FoxG1* heterozygous (*n* = 25) animals. Both wildtype and *Gad2* null animals spent significantly more time in the novel arm compared to the familiar arms [*p* = 8.39×10(−30)**** for WT and *p* = 6.21×10(−15)**** for *Gad2* null]. In the ASD model background, time spent in the familiar and novel arms was comparable for both wildtype (*p* = 0.159) and *Gad2* nulls (*p* = 0.431). Time spent in the novel arm was also comparable with *Gad2* mutation in both wildtype and ASD model backgrounds (*p* = 0.289 for wildtype and *p* = 0.193 for the ASD model). These results suggest that, unlike social behavior, the *Gad2* mutation does not affect novel space preference in either wildtype or ASD model animals, indicating that GABAergic development is not centrally involved in this behavior. The raw data points for 1.95, 1.97 (**C**, WT), 1.79, 1.91, 1.93 (**C**, ASD) and 188.9 (**D**, WT/*Gad2*, Nov) are not shown. Data are mean ± SEM, p values are from two-tailed *t*-tests.

These results raised the possibility that the social behavioral deficit observed in the ASD model at 6 weeks (Miyoshi et al., 2021) could also be properly established earlier at 3 weeks. To test this, we conducted a three-chamber social behavioral assay for the ASD model and the control littermate wildtypes at 3 weeks (P21), following the same protocol as our study for 6-week-old animals (Miyoshi et al., 2021). After 10 min of habituation in the arena (Figure 4A), sociability was assessed by offering the choice between a chamber containing a stranger mouse and an empty chamber (Figure 4A). In the subsequent session, a second stranger mouse was placed in the previously empty chamber, giving the animals the choice between a familiar and a novel mouse to assess social novelty (Figure 4A). We found that wildtype animals spent more time in the chamber containing a stranger mouse during the sociability session and preferred to spend time in the chamber with the novel mouse over the familiar mouse during the social novelty session (Figure 4B). These data suggest that sociability and social novelty are established in wildtype mice by 3 weeks of age. In contrast, the ASD model spent comparable amounts of time in the chamber with the stranger mouse and the empty chamber during the sociability session, and also showed no preference between the familiar and novel mice during the social novelty session (Figure 4B), resulting in significantly lower social scores (Figure 4C). Furthermore, the time spent in the center chamber during the social novelty session was significantly increased in the ASD model compared to control animals, suggesting that the ASD model avoids interacting with other mice (Figure 4B). These data indicate that sociability is impaired in the ASD model by 3 weeks and that this deficit persists through 6 weeks into adulthood.

Given the observed difference between novel space and social preference at 3 weeks, we next examined the role of GABAergic neuron development in novel space preference. We used a transgenic mutant for *Gad2*, a gene encoding a synthetic enzyme for the GABA neurotransmitter. In the three-chamber social assay, reduced GABAergic tone via *Gad2* null mutation decreased the sociability score in wildtype animals and exacerbated ASD-like social impairments in the ASD model (Miyoshi et al., 2021). To investigate the impact of reduced GABAergic tone on novel space preference, we similarly combined *Gad2* mutant animals with the ASD model and conducted the V-Y assay at 6 weeks (P43-45, Figure 4D). Specifically, we crossed *FoxG1-LacZ*; *Gad2-null* double-heterozygous males with *Gad2-null* heterozygous females to generate the experimental animals. Similar to their wildtype littermates, *Gad2* mutants spent significantly more time in the novel arm compared to the familiar arms (Figure 4D). Furthermore, the time spent in the novel arm was comparable between wildtype and *Gad2* null animals, suggesting that reduced GABAergic tone has no impact on novel space preference. Similarly, when we compared the ASD model (*FoxG1* heterozygous) with the ASD model carrying reduced GABAergic tone (*FoxG1* heterozygous and *Gad2* null), we found no significant differences in novel space preference. Consistent with our earlier findings (Figure 2E), the time spent in the novel and familiar arms was comparable in the ASD model, and this trend was similar in the *Gad2* null ASD model (Figure 4D). In addition, the time spent in the novel arm was also comparable between these two genotypes. These data strongly suggest that a reduction in GABAergic tone during development does not impact novel space preference, even in the ASD model. Altogether, our findings demonstrate that novel space preference in the ASD model undergoes a unique pattern of developmental regression that occurs independently of overall GABAergic tone and is distinct from the development of social behavior circuits.

## Discussion

In summary, we developed a novel V-Y maze assay suitable for detecting novel space preference and exploratory behavior in both juvenile and adult mouse models. We demonstrated that novel space preference is established during early juvenile stages but subsequently regresses by postnatal 6 weeks in our ASD mouse model. This regression occurs independently of GABAergic circuit development, unlike the social behavior impairments observed in this model.

We modified the classic Y-maze to develop a novel space preference V-Y assay by blocking one arm of the Y-maze during the initial half of the assay. Through this approach, we were able to directly compare novel space preference between wildtype and ASD model mice. In addition to assessing novel space preference, this assay enabled us to investigate exploratory behavior by analyzing wall-seeking tendencies based on how often the animals’ heads were oriented toward the wall. Furthermore, we found that this assay is well-suited for studying developmental processes in juvenile mice. Specifically, we analyzed postnatal 2-and 3-week-old animals and demonstrated that novel space preference is established by 3 weeks of age but subsequently regresses by 6 weeks in our ASD model.

In our V-Y maze assay, we focused on novel space preference, but how is working memory affected in the ASD model? In a previous study, we found that the *FoxG1* haploinsufficiency ASD model displays working memory deficits using an 8-arm maze with water droplets as a reward (Miyoshi et al., 2021). Interestingly, while social behavior impairments were either exacerbated or ameliorated depending on the modulation of GABAergic tone, working memory remained unaffected. It is possible that the novel space preference phenotype observed in this study may correlate with the working memory of the animals. Similarly, preference for a novel object was also found to be impaired in the adult *FoxG1* haploinsufficiency model (Younger et al., 2022) using a *Cre* knock-in *FoxG1* allele (Hebert and McConnell, 2000). Other ASD models, including those with syndromic gene mutations, have been shown to exhibit spatial and/or working memory deficits (Berkowicz et al., 2016; Boku et al., 2018; Lim et al., 2017; Nakamura et al., 2021; Rendall et al., 2016). Therefore, it may be reasonable to investigate the developmental trajectory of working memory and novel object recognition in our ASD model at postnatal week 3 to determine whether these abilities are initially acquired but subsequently regress by week 6. In terms of spatial preference, while ASD model animals exhibited a similar environmental preference to wildtypes, interestingly, this preference was initially suppressed but became more pronounced and comfortable over time in the ASD model (Narita et al., 2024). These findings align with observations in human ASD individuals, who may initially struggle to process spatial information but can still distinguish their surroundings and identify comfortable spaces (Smith, 2015).

Which brain region regulates novel space preference? Novelty detection and association are processed by hippocampal networks (Knight, 1996; Kumaran and Maguire, 2007). Spatial navigation in a novel environment is primarily handled by hippocampus, with the posterior hippocampal regions showing a greater response to environmental novelty than to object novelty (Kaplan et al., 2014). Moreover, theta rhythms in prefrontal regions are thought to facilitate the integration of new information into memory through communication with the hippocampus (Chrastil et al., 2022). Interestingly, ASD patients who experience navigation difficulties often retain intact spatiotemporal memory but exhibit impairments in upstream multisensory information processing (Laidi et al., 2023). Additionally, language and spatial working memory are coded separately in the brain, which may explain why some ASD patients show language impairments while visual memory and processing speed remain unaffected (Hill et al., 2015). In our previous study, we identified a transient increase in the excitatory/inhibitory (E/I) ratio in the medial prefrontal cortex (mPFC) of our ASD model at 2 weeks (P14). To address this, we bilaterally transplanted embryonic GABAergic neuronal precursors into the P7 mPFC, aiming to enhance GABA tone within this region. This intervention ameliorated the social impairments observed in the ASD model. Conversely, *Gad2* mutation, which globally reduced GABAergic tone from early development, further exacerbated the social behavioral impairments in the ASD model (Miyoshi et al., 2021). In the present study, using the same *Gad2* manipulation, we found that the reduction of GABAergic tone had no effect on novel space preference, as assessed by our V-Y assay (Figure 4D). We attribute these findings to differences in the requirements for GABAergic tone. However, it is also possible that social behavior and novel space preference have distinct thresholds for GABA. Alternatively, the brain circuits underlying these behaviors may be differentially affected by a similar decrease in GABA tone due to the *Gad2* mutation.

In this study, we utilized the *FoxG1* heterozygous null ASD model; however, how can our findings be generalized to ASD? This model exhibits impairments in both sociability and social novelty, characterized by avoidance of stranger animals (Miyoshi et al., 2021). Additionally, *FoxG1* heterozygous null mice display increased activity levels (open field), anxiolytic behavior (elevated plus maze), reduced working memory (8-arm radial maze), and decreased gamma EEG power in the mPFC (Miyoshi et al., 2021). Furthermore, studies using Cre knock-in *FoxG1* heterozygous animals have demonstrated defects in novel object recognition and fear memory, along with increased anxiety in the open field (Younger et al., 2022). Abnormal locomotion and impairments in contextual fear conditioning were first identified in tTA knock-in *FoxG1* heterozygous animals (Shen et al., 2006). Thus, *FoxG1* heterozygous mice exhibit a characteristic behavioral profile, including these deficits in addition to social impairments. Moreover, *FOXG1* dysregulation has been linked not only to ASD but also to neuropsychiatric disorders such as schizophrenia (Won et al., 2016). It would be highly informative to assess how other established syndromic ASD mouse models, as well as valproic acid-induced ASD models (Chaliha et al., 2020; Nicolini and Fahnestock, 2018), perform in our V-Y assay. Of particular interest is the *Mecp2* mutant model, to determine whether it exhibits a regression in scores similar to the regression observed in Rett syndrome patients (Sztainberg and Zoghbi, 2016).

In our V-Y maze assay, the 6-week-old ASD model not only shows diminished novel space preference but also exhibits reduced exploratory behavior, as indicated by a decrease in wall-seeking. It has been reported that ASD individuals exhibit reduced novel space preference, are less likely to explore environments thoroughly, and are more likely to revisit previously explored locations (Smith, 2015). We propose that the V-Y maze assay is a suitable tool for simultaneously analyzing novel space preference and exploratory behavior in ASD models (Bourgeron, 2015; Del Pino et al., 2018; Fuccillo, 2016; Golden et al., 2018; Mullins et al., 2016; Sztainberg and Zoghbi, 2016; Takumi et al., 2020). Here, we demonstrate that social behavior and novel space preference are regulated by independent brain networks, with only the former depending on proper GABAergic circuit development (Fishell and Kepecs, 2020; Kupferschmidt et al., 2022; Lunden et al., 2019; Miyoshi, 2019; Tang et al., 2021). We propose that distinct approaches must be taken to address both social behavior impairments and novel space preference/exploration deficits in the treatment of individuals with ASD.

## Data Availability

The original contributions presented in the study are included in the article/supplementary material, further inquiries can be directed to the corresponding author.
